# Severe morbidity and hospital-based mortality from Rift Valley fever disease between November 2017 and March 2020 among humans in Uganda

**DOI:** 10.1186/s12985-024-02377-z

**Published:** 2024-05-03

**Authors:** Zacchaeus Anywaine, Christian Hansen, George M. Warimwe, Ggayi Abu-Baker Mustapher, Luke Nyakarahuka, Stephen Balinandi, Alex Riolexus Ario, Julius J. Lutwama, Alison Elliott, Pontiano Kaleebu

**Affiliations:** 1 Department of Clinical Research, London School of Hygiene and Tropical Medicine, London, UK; 2 Medical Research Council, Uganda Virus Research Institute and London School of Hygiene and Tropical Medicine Uganda Research Unit, Plot 51 - 59 Nakiwogo Road, P. O. Box 49, Entebbe, Uganda; 3 MRC International Statistics and Epidemiology Group, London School of Hygiene and Tropical Medicine, London, UK; 4 Centre for Tropical Medicine and Global Health, University of Oxford, Oxford, UK; 5 KEMRI-Wellcome Trust Research Programme, Kilifi, Kenya; 6 Department of Arbovirology, Emerging and Re-emerging Infectious Diseases, Uganda Virus Research Institute, Entebbe, Uganda; 7 Department of Biosecurity, Ecosystems and Veterinary Public Health, College of Veterinary Medicine, Animal Resources and Biosecurity, Makerere University, Kampala, Uganda; 8 National Institute of Public Health, Ministry of Health, Kampala, Uganda

## Abstract

**Background:**

Rift Valley fever (RVF) is a zoonotic viral disease of increasing intensity among humans in Africa and the Arabian Peninsula. In Uganda, cases reported prior to 2016 were mild or not fully documented. We report in this paper on the severe morbidity and hospital-based mortality of human cases in Uganda.

**Methods:**

Between November 2017 and March 2020 human cases reported to the Uganda Virus Research Institute (UVRI) were confirmed by polymerase chain reaction (PCR). Ethical and regulatory approvals were obtained to enrol survivors into a one-year follow-up study. Data were collected on socio-demographics, medical history, laboratory tests, potential risk factors, and analysed using Stata software.

**Results:**

Overall, 40 cases were confirmed with acute RVF during this period. Cases were not geographically clustered and nearly all were male (39/40; 98%), median age 32 (range 11–63). The median definitive diagnosis time was 7 days and a delay of three days between presumptive and definitive diagnosis. Most patients (31/40; 78%) presented with fever and bleeding at case detection. Twenty-eight (70%) cases were hospitalised, out of whom 18 (64%) died. Mortality was highest among admissions in regional referral (11/16; 69%) and district (4/5; 80%) hospitals, hospitalized patients with bleeding at case detection (17/27; 63%), and patients older than 44 years (9/9; 100%). Survivors mostly manifested a mild gastro-intestinal syndrome with nausea (83%), anorexia (75%), vomiting (75%), abdominal pain (50%), and diarrhoea (42%), and prolonged symptoms of severe disease including jaundice (67%), visual difficulties (67%), epistaxis (50%), haemoptysis (42%), and dysentery (25%). Symptom duration varied between two to 120 days.

**Conclusion:**

RVF is associated with high hospital-based mortality, severe and prolonged morbidity among humans that present to the health care system and are confirmed by PCR. One-health composite interventions should be developed to improve environmental and livestock surveillance, prevent infections, promptly detect outbreaks, and improve patient outcomes.

**Supplementary Information:**

The online version contains supplementary material available at 10.1186/s12985-024-02377-z.

## Background

Rift Valley fever (RVF) is caused by the Rift Valley fever virus (RVFV) which is an enveloped phlebovirus that infects animals and humans [1] causing a spectrum of disease manifestations. Before 2000, outbreaks were infrequent and cases were mostly mild, but over the last two decades the disease has occurred with increased intensity. Cases characterised by hepatitis, blindness, abortions, encephalitis, haemorrhage, renal failure, and death are increasingly reported [2]. Between 1931 and 1974 a single fatal case of RVF was reported in humans [3]. In 2015, a systematic review by Nanyingi et al. reported a mortality of 0.3 to 5.9% between 1975 and 1999, and 27.7–44.7% between 2000 and 2012 [4]. In Uganda, the virus was first isolated from mosquitoes in 1944 [5] and human field outbreaks were first reported between 1960 and 1968 when 16 cases from three outbreaks were diagnosed from villages near the then Yellow Fever Research Institute in Entebbe, currently the Uganda Virus Research Institute (UVRI) [6]. For nearly 50 years till 2016, there were no cases reported in the country. In 2016, an outbreak involving three people occurred in the South-Western district of Kabale, and in all these outbreaks no human mortality was reported [7]. Since 2017 the country has experienced several outbreaks of RVF in humans. Cases have been reported as presenting with the general febrile/flu-like, gastrointestinal, and haemorrhagic symptoms and a case fatality rate of 42%, however, there was no data on hospitalisation, duration of symptoms, where and in whom the mortality occurred, thus difficult to fully evaluate the intensity of the disease [8]. In this paper, we provide detailed information on the hospital-based mortality, prolonged and severe morbidity, and long-term recovery of survivors from RVF in Uganda.

## Methods

### Study design and settings

This study collected cross-sectional and prospective cohort data from patients. Between November 2017 and March 2020, alerts of human cases of febrile illness were reported to the Uganda Ministry of Health from several health facilities and districts in Uganda [9]. Using the national case definition for RVF (Additional file 1) [10], cases were identified by the National Rapid Response Teams (NRRT) and UVRI. This study report is limited to March 2020 when recruitment to the long-term follow up study was stopped due to cost, yet more cases (not included in this report) have been documented beyond this date. We defined severe morbidity based on whether the patient’s condition required hospitalisation [11].

### Collection of UVRI and NRRT surveillance data

Blood specimens were obtained from suspected cases by the health workers or the UVRI surveillance team and sent to the UVRI for laboratory confirmation. The UVRI is the national reference laboratory for the diagnosis of viral haemorrhagic fevers (VHFs). The specimens were tested for VHF viruses using Enzyme Linked Immunosorbent Assay Immunoglobulin M (ELISA IgM), ELISA IgG, and polymerase chain reaction (PCR) tests and found to be positive only for RVF. ELISA IgM/IgG and PCR tests were conducted as previously described and adapted for RVF in human samples [12, 13]. Briefly, for RVF IgG testing, 96-well Microtiter plates (Thermo Electron Corporation, Milford, MA) were coated overnight at 4^o^C with an RVF antigen in the upper half and a mock antigen in the lower half, followed by addition of heat and detergent inactivated human sera prediluted to 1:100, 1:400, 1:1600 and 1:6400, including the negative and positive controls. Plates were incubated for 1 h in a humidified chamber at 37^o^C, washed 3 times with 250 µl of buffer (PBS containing 0.1% Tween-20 v/v), and 100 µl of a mouse anti-human IgG (Accurate Chemicals, NY, USA) in a 1:1000 dilution added with further incubation for 1 h at 37 °C. Plates were washed, incubated for 30 min at 37 °C with 100 µL/well of 2,2′-azinobis-(3-ethylbenzothiazoline-6-sulfonic acid) (ABTS) substrate (KPL, Gaithersburg, MD), and read spectrophotometrically at 490 nm. A sum optical density (ODSum) for each test serum was obtained by adding the differences between the OD values of the control antigen-coated wells from their corresponding RVF-antigen-coated wells. A positive diagnosis for RVF IgG in the respective test serum was scored if its ODSum was ≥ 1.00.

For IgM testing, procedures such as sample and reagent volumes, washing and incubation steps, were performed as for the IgG testing procedure above, except in this case, plates were precoated with a goat anti-human IgM capture antibody (Sera care Life Sciences, MA, USA), followed by an overnight incubation at 4^o^C, before the addition of human sera, positive and negative control samples (also prediluted as in the case of IgG testing above). After a 1-hour incubation period at 37^o^C in a humidified chamber, and a washing step, an RVF antigen was added in the upper part of the plate and a mock antigen in the lower part, which was then followed by the addition of a mouse anti-human RVF IgM antibody (Thermo Scientific, IL, USA) followed by the ABTS substrate (KPL, Gaithersburg, MD) and read spectrophotometrically at 490 nm. Unlike for IgG, positive IgM samples were those with ODSum ≥ 0.45.

Quantitative real time PCR (qrtPCR) was conducted as previously described [7, 13]. In summary, viral RNA was isolated using MagMax magnetic bead kit (Life Technologies, Carlsbad, CA) according to the manufacturers protocol. qrtPCR was conducted with established primer and probe sets for the RVFV L segment.

The UVRI and NRRT collected information on socio-demographics such as age, sex, residence (district, subcounty, parish, village), and occupation; medical data on symptoms, dates of (illness onset, case report, laboratory specimen reception, and testing), hospitalisation, disease outcome (alive or died), and laboratory test results; and risk of contact with livestock. The case record lists from the UVRI and NRRT were merged for the period between November 2017 and March 2020. Survivors were contacted after full recovery from the acute illness, consented, and enrolled into a 1-year follow-up study at the MRC/UVRI and LSHTM Uganda Research Unit clinic in Masaka, Southwestern Uganda.

### Enrolment and follow up of survivors

At enrolment participant demographics, exposure to RVF risk factors, medical history and physical examination, blood specimens for full blood count (FBC) and liver function tests (LFTs), serum, plasma, and peripheral blood mononuclear cells (PBMCs) were obtained. Participants were followed monthly to obtain clinical information, whereas FBC, LFTs, serum, plasma and PBMCs were done every three months.

Study data were captured on case record forms and entered into OpenClinica database. The data from the combined UVRI/NRRT log was further merged with the data from the OpenClinica database. Data were analyzed using Stata statistical software version 17.0. We excluded patients with positive anti-RVF IgG and negative IgM or PCR results. Time to ascertainment of diagnosis was calculated as median number of days, and occurrence of events/outcomes (clinical signs and symptom, risk factors, hospitalization, and/or death) presented as proportions. We investigated whether the distribution of assessed parameters overlapped among survivors and non-survivors using the Mann-Whitney test, and the association between age-group and mortality using a Fisher’s exact test. Haematological and biochemistry parameters were summarized descriptively using box plots.

## Results

### Study schema

**Fig. 1:**
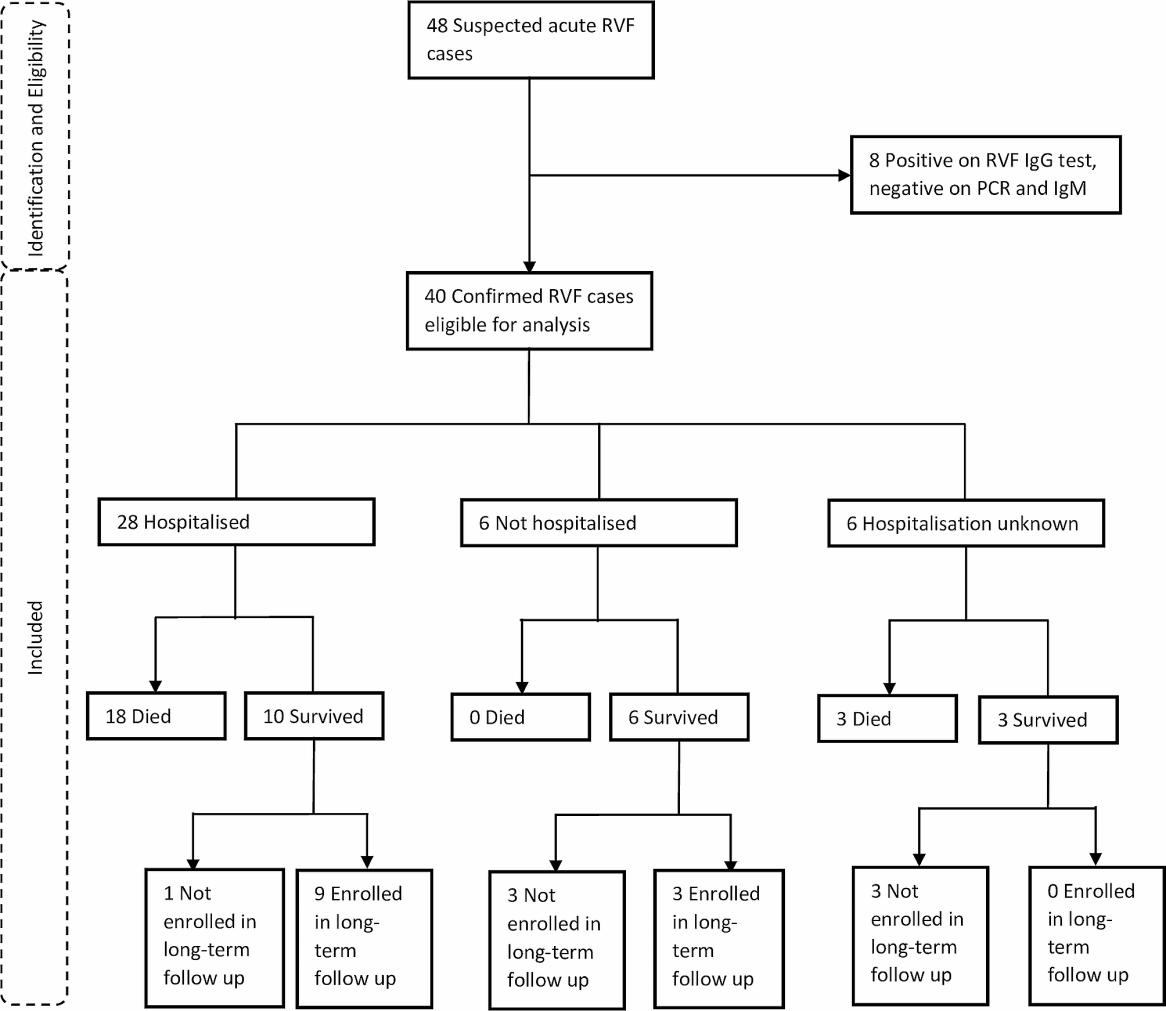
Flow diagram of RVF cases between November 2017 and March 2020

The flow diagram of cases in this study is shown in Fig. 1. Forty-eight (48) suspected acute RVF cases were reported to the UVRI/NRRT between November 2017 and March 2020. Eight cases were RVF IgG positive but PCR and IgM negative and were excluded. Forty (40) confirmed acute cases were eligible for this analysis. Twenty-eight (70%) eligible cases were hospitalised, out of whom 18 (64%) died and 10 survived. Nine out of the ten hospitalised survivors were enrolled into long-term follow. Six eligible cases were survivors without a history of hospitalisation and three of them were enrolled in the long-term follow up. The hospitalisation status of six eligible cases was not recorded, and of these three died and three survived, and none was enrolled in the long-term follow up study. The spatial-temporal distribution of cases in this outbreak is published in a paper by Nyakarahuka et al. 2023 [8].

### Epidemiological clustering of cases

**Fig. 2:**
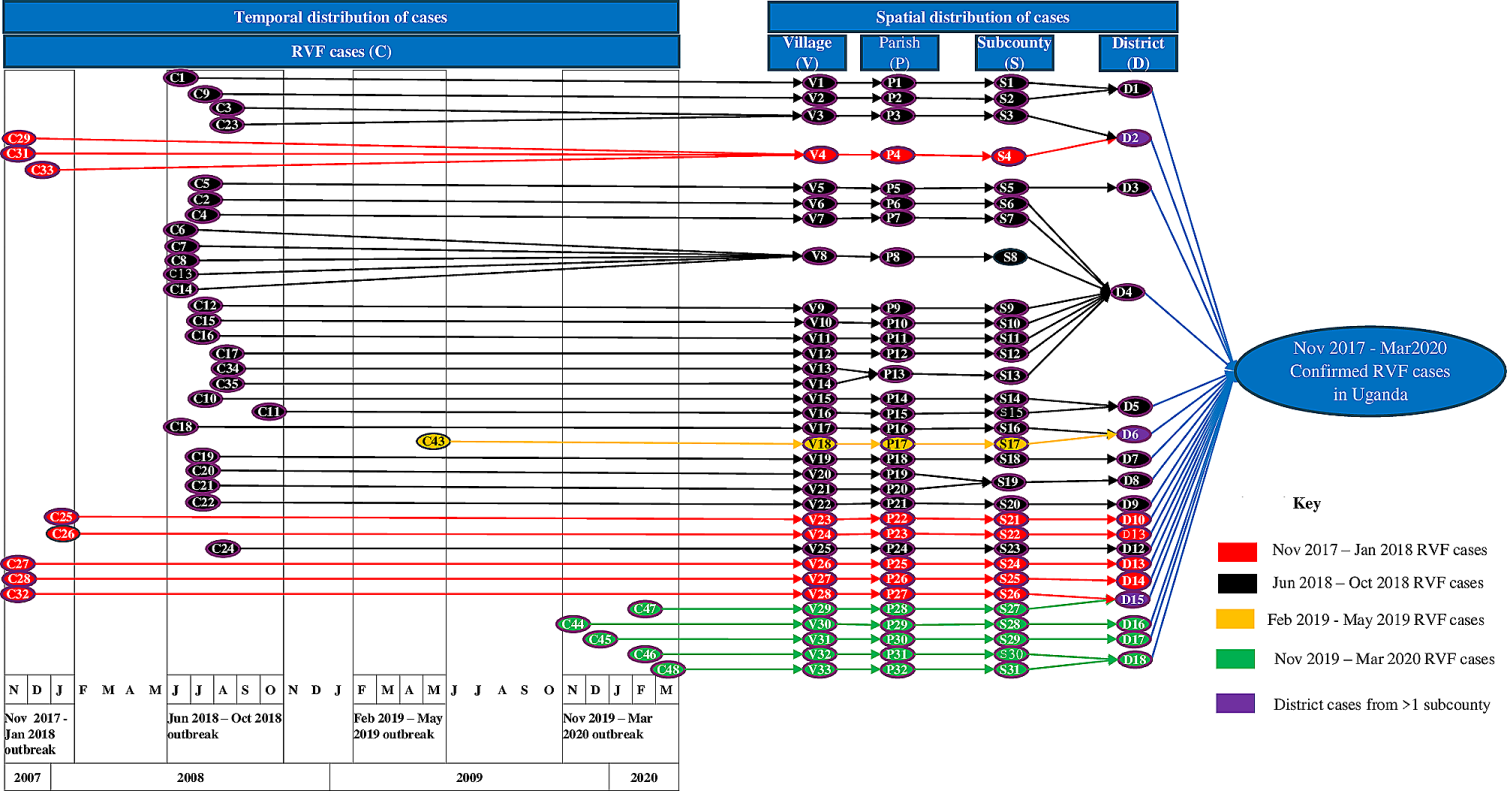
Epidemiological cluster tree for November 2017 to March 2020 RVF outbreak in Uganda

The epidemiological cluster tree in Fig. 2 shows that cases occurred in four temporal clusters: November 2017 to January 2018, June 2018 to October 2018, February 2019 to May 2019, and November 2019 to March 2020. Generally, no clear spatial clustering of cases is seen at village, parish, and subcounty units. Only three villages (V3, V4 and V8) had more than one case reported indicating no clustering at small geographical units (villages). One parish (P13) had cases reported from two villages and one subcounty (S19) from two different parishes. One district (D4) had cases in several sub counties during the same outbreak cluster whereas ten districts (D3, D7, D9, D10, D11, D12, D13, D14, D16, D17) reported a single case each. Three districts (D2 (black & red), D6 (black & yellow), D15 (red & green)) had cases reported from two different outbreak clusters which are distinguished by the different colour codes.

### Demographic and clinical characteristics

Most suspected cases were male (44/48; 92%) (Table 1). The median age of all suspected cases was 32 (range 10–67) years, higher among non-survivors than survivors (36 versus 29 years, *p* = 0.01 on a Mann-Whitney test). The median presumptive case diagnostic time was 4 days, definitive diagnosis time 7 days, laboratory sample delivery time 3 days, and laboratory turnaround time < 1 day. There was a delay of three days between presumptive and definitive diagnosis. This delay was higher among survivors than non-survivors (4 days versus 1 day, *p* = 0.005 on a Mann-Whitney test). The definitions of presumptive and definitive diagnostic time are indicated in the legend section of Table 1.

Eighty-three percent (40/48; 83%) of cases were confirmed with acute RVFV infection and more than half (21/40; 53%) died. The mortality was highest in regional referral (RRH) (11/16; 69%) and district (4/5; 80%) hospitals. Fever and bleeding were the commonest manifestation at case detection in 96% (27/28) of hospitalised patients and more than half (17/27; 63%) of the hospitalised patients that presented with bleeding died.

**Table 1:** Study population characteristics

Characteristic		Category	Parameter estimate		
			Survivors	Non-survivors	Overall/Total
All RVF suspected cases reported between November 2017 and March 2020 (*n* = 48)	Age	Median (range), years	29 (10–67)	36 (18–63)	32 (10–67)
	Sex	Male, n (%)	24 (55)	20 (45)	44 (100)
		Female, n (%)	3 (75)	1 (25)	4 (100)
	Presumptive case diagnostic time	Median (range), days	8.5 (0–32)	2.5 (0–14)	4 (0–32)
	Laboratory sample delivery time	Median (range), days	4 (1-7)	1 (0–8)	3 (0–8)
	Laboratory turnaround time	Median (range), days	0 (0–1)	0 (0–1)	0 (0–1)
	Definitive case diagnostic time	Median (range), days	13 (3-36)	6 (1-15)	7 (1-36)
	Presumptive to Definitive case diagnostic time	Median (range), days	4 (1-7)	1 (0–8)	3 (0–8)
	IgG positive	Yes, n (%)	14 (100)	0 (0)	14 (100)
	PCR/IgM positive	Yes, n (%)	19 (48)	21 (52)	40 (100)
		No, n (%)	8 (100)	0 (0)	8 (100)
RVF confirmed positive cases between November 2017 and March 2020 (*n* = 40)	Age	Median (range), years	27 (11-41)	36 (18–63)	32 (11–63)
	Age group	< 25 years, n (%)	7 (78)	2 (22)	9 (100)
		25–44 years, n (%)	12 (55)	10 (45)	22 (100)
		> 44 years, n (%)	0 (0)	9 (100)	9 (100)
	Sex	Male, n (%)	19 (49)	20 (51)	39 (100)
		Female, n (%)	0 (0)	1 (100)	1 (100)
	Hospitalisation status of positive cases	Yes, n (%)	10 (36)	18 (64)	28 (100)
		No, n (%)	6 (100)	0 (0)	6 (100)
		Unknown, n (%)	3 (50)	3 (50)	6 (100)
	Health facility	Regional referral hospitals, n (%)	5 (31)	11 (69)	16 (100)
		District hospitals, n (%)	1 (20)	4 (80)	5 (100)
		Private hospitals, n (%)	2 (67)	1 (33)	3 (100)
		Health Centre IIIs, n (%)	2(50)	2 (50)	4 (100)
		Community case, n (%)	6 (100)	0 (0)	6 (100)
		Unknown hospitalisation status, n (%)	3 (50)	3 (50)	6 (100)
	Bleeding at time of case detection among hospitalized	Yes, n (%)	10 (37)	17 (63)	27 (100)
		No, n (%)	0 (0)	0 (0)	0 (0)
		Unknown, n (%)	0 (0)	1 (100)	1 (100)
	Fever at time of case detection among hospitalized	Yes, n (%)	10 (37)	17 (63)	27 (100)
		No, n (%)	0 (0)	0 (0)	0 (0)
		Unknown, n (%)	0 (0)	1 (100)	1 (100)

Presumptive case diagnostic time - refers to time between symptom onset and clinical diagnosis; Laboratory sample delivery time - refers to time between clinical diagnosis to delivery of samples in the laboratory for testing; Laboratory turnaround time – refers to time between reception of sample in the laboratory and communication of test results to the health worker/facility; Definitive case diagnostic time – refers to time between symptom onset and laboratory case confirmation including communication of results to health worker/facility; Presumptive to Definitive case diagnostic time – refers to time between making a presumptive case diagnosis and definitive case diagnosis

Regional referral hospitals- include Arua and Mbarara hospitals; District hospitals – include Kawolo, Kiboga, Kilembe, Moyo and Nagulu hospitals; Private hospitals – include Ibanda, Mengo and Nakasero hospitals; Health Centre (HC)III – include Bijubuli, Bussi, Rwebisengo and Sanga HCIIIs

**Fig. 3:**
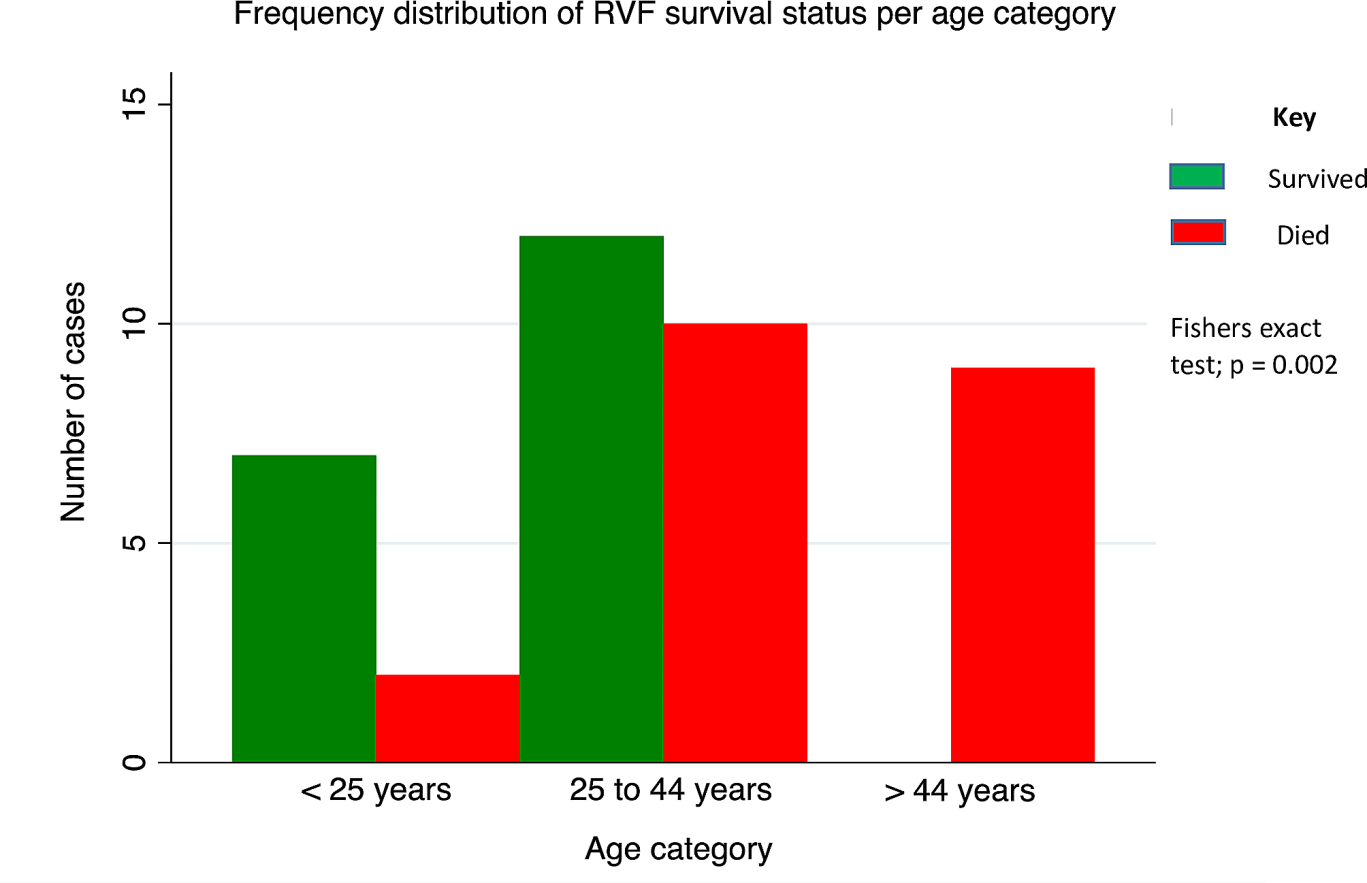
Frequency distribution of RVF survival status per age category

Using the broader medical subject heading (MeSH) age categories (Fig. 3), the trend in mortality increased with age-group. Mortality was lowest (2/9; 22%) among the young (children, adolescents, and young adults), higher (10/22; 46%) in adults 25–44 years, and all patients (9/9; 100%) above 44 years died. There was a statistically significant association between age-group and mortality (Fisher’s exact test; two-sided *p* = 0.002).

### Clinical manifestations of RVF among survivors

Among survivors (Table 2), gastro-intestinal manifestations including nausea (83%), anorexia (75%), vomiting (75%), abdominal pain (50%), and diarrhoea (41.7%) were the most common presentation. Patients reported symptoms of severe disease including jaundice (67%), visual impairment (67%), epistaxis (50%), haemoptysis (42%), haematemesis (42%), bleeding gums (25%), and dysentery (25%). Neurological manifestations including loss of consciousness (33%), convulsions (17%), and neck pain (8%) were the least common. Symptom duration varied between two to 120 days.

**Table 2:** Clinical manifestations of RVF among survivors enrolled in the long-term follow-up study

Clinical syndrome/symptoms	Number (%)	Duration of symptom; median (range), days
	Overall *N* = 12	
General febrile/influenza-like syndrome		
Fever	8 (67)	18 (2–99)
Headache	8 (67)	26 (7–120)
Muscle pains	5 (42)	21 (3–120)
Joint pains	6 (50)	7 (3–120)
Gastro-intestinal syndrome		
Anorexia	9 (75)	7 (3-42)
Nausea	10 (83)	7 (2-42)
Vomiting	9 (75)	6 (2-42)
Abdominal pain	6 (50)	22 (8–120)
Diarrhoea	5 (42)	26 (4–62)
Constipation	2 (17)	7 (7-7)
Hepatic syndrome		
Jaundice	8 (67)	15 (5–60)
Visual syndrome		
Visual difficulties	8 (67)	14 (3–60)
Neurological syndrome		
Neck pain	1 (8)	2 (2-2)
Convulsions	2 (17)	3 (1-4)
Loss of consciousness	4 (33)	4 (2-7)
Haemorrhagic syndrome		
Bleeding from gums	3 (25)	7 (3-7)
Haemoptysis	5 (42)	7 (2-30)
Haematemesis	5 (42)	14 (7-14)
Epistaxis	6 (50)	14 (4–90)
Dysentery	3 (25)	7 (3-30)

**Fig. 4:**
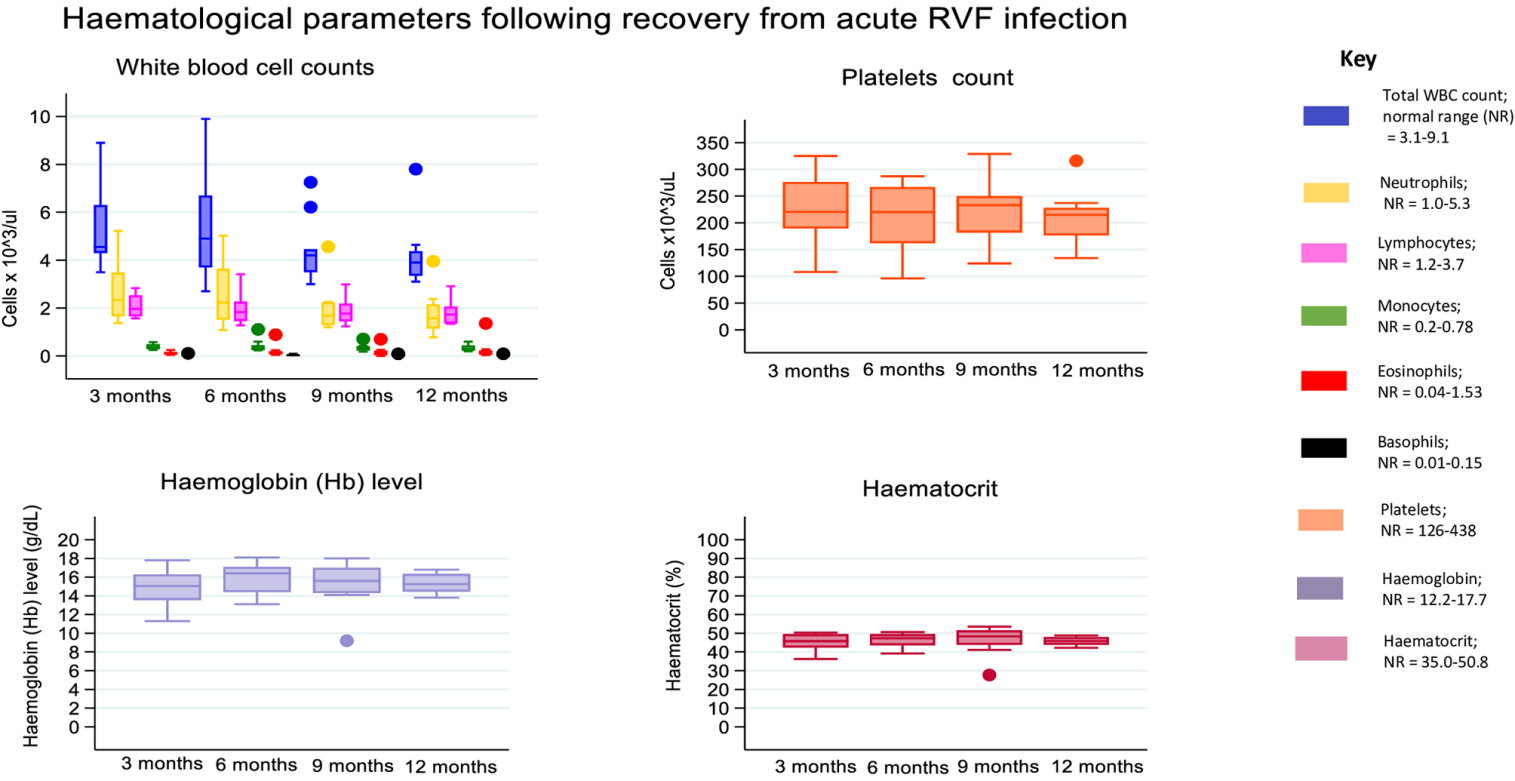
Full blood count parameters among RVF survivors followed for one year

**Fig. 5:**
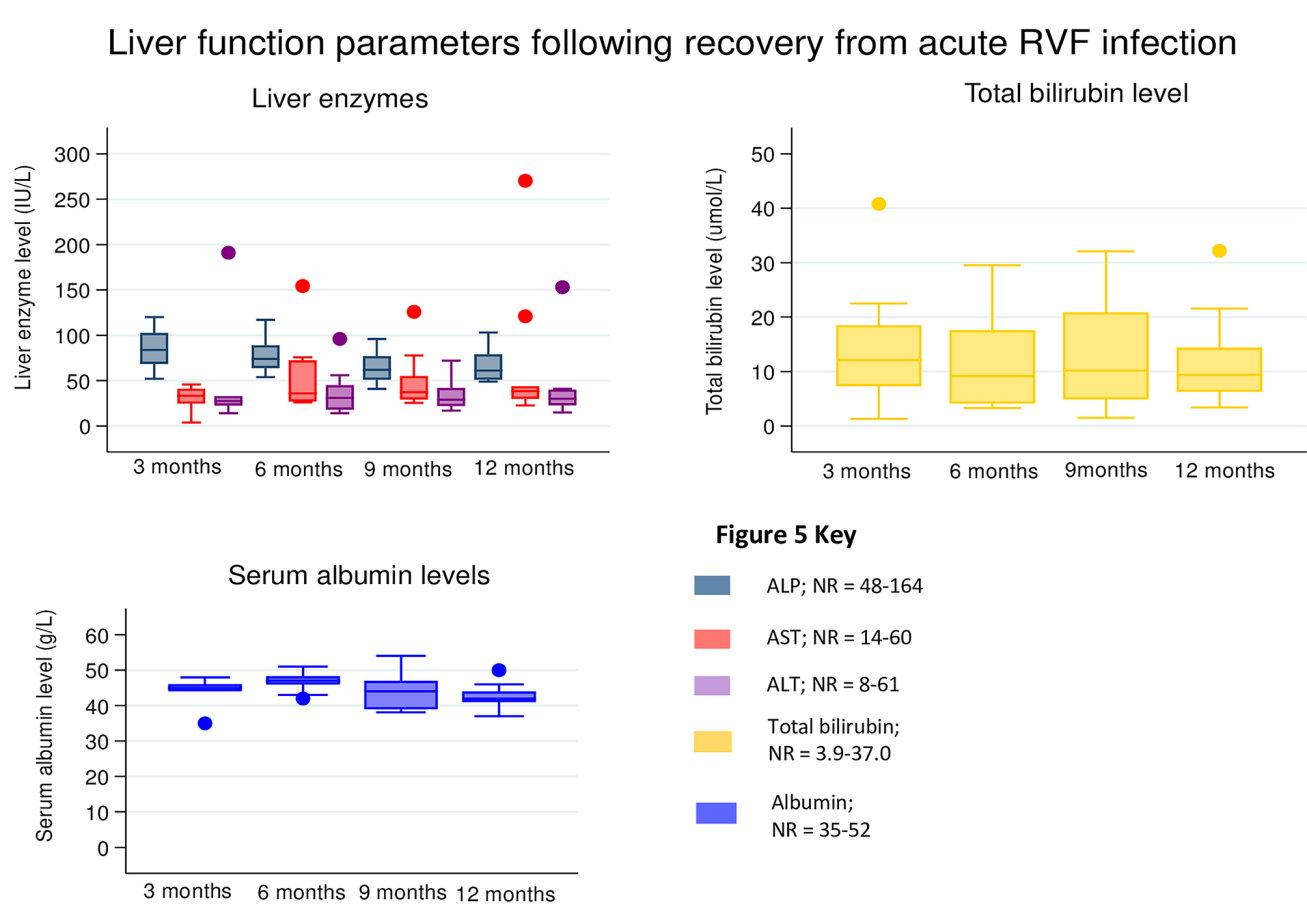
Liver function parameters among RVF survivors followed for one year

Survivors were enrolled three months following a definitive diagnosis of RVF by the UVRI and by this time the total white blood cell counts, and differential cell counts (neutrophils, lymphocytes, eosinophils, basophils, monocytes, and platelets), haemoglobin/haematocrit (Fig. 4), liver enzymes (alkaline phosphatase (ALP), aspartate aminotransferase (AST), and alanine amino transferase (ALT)), total bilirubin, and albumin levels (Fig. 5), had returned to normal range, and were maintained till month 12.

### RVF risk factors among survivors

Only men were enrolled in the long-term follow up study (Table 3). The consumption of animal products (milk, ghee, and meat) was the most common (10/12; 83%) potential risk factor reported. One survivor reported consumption of a fetus delivered following caesarean section on a cow and another consumption of wild game meat (squirrel). Two (17%) patients reported sleeping near or shared shelter with animals (Fig. 3a), there was low mosquito net use (2/12; 17%), and residences were remotely located with poor access by road (Fig. 3b – d) or close (median 1.5, range (0.5 to 10) kilometres) to permanent water bodies which are mosquito breeding habitats.

**Table 3:** Potential risk factors to RVF infection among survivors enrolled in long-term follow-up study

Characteristic	Category	RVF survivors included in follow up study (*n* = 12)
Age	Median (range), years	32.5 (19–48)
Sex	Male, n (%)	12 (100)
	Female, n (%)	0 (0)
Occupation	Farmer, n (%)	7 (58)
	Herdsman, n (%)	3 (25)
	Trader, n (%)	2 (17)
Activities in month preceding sickness	Contact with sick animals, n (%)	5 (42)
	Abortions in livestock, n (%)	2 (17)
	Assisted animal birth, n (%)	3 (25)
	Disposed aborted foetus, n (%)	2 (17)
	Touched dead animals, n (%)	3 (25)
	Consumed livestock products (milk, ghee and meat including delivered fetuses), n (%)	10 (83)
	Hunted wild game (Squirrel), n (%)	1 (8)
	Nursed patient with febrile illness, n (%)	3 (25)
	Death of family member from suspicious illness, n (%)	3 (25)
Animals interacted with	Sheep, n (%)	4 (44)
	Goats, n (%)	3 (33)
	Cattle, n (%)	3 (33)
	Chicken, n (%)	2 (22)
Location of residence	Sleep near or shared shelter with animals, n (%)	2 (17)
	Distance from water body, median (range), kms	1.5 (0.5–10)
Mosquito net use	No mosquito net use, n (%)	2 (17)
Travel history	Travelled to more than one district, n (%)	5 (42)

**Fig. 6:**
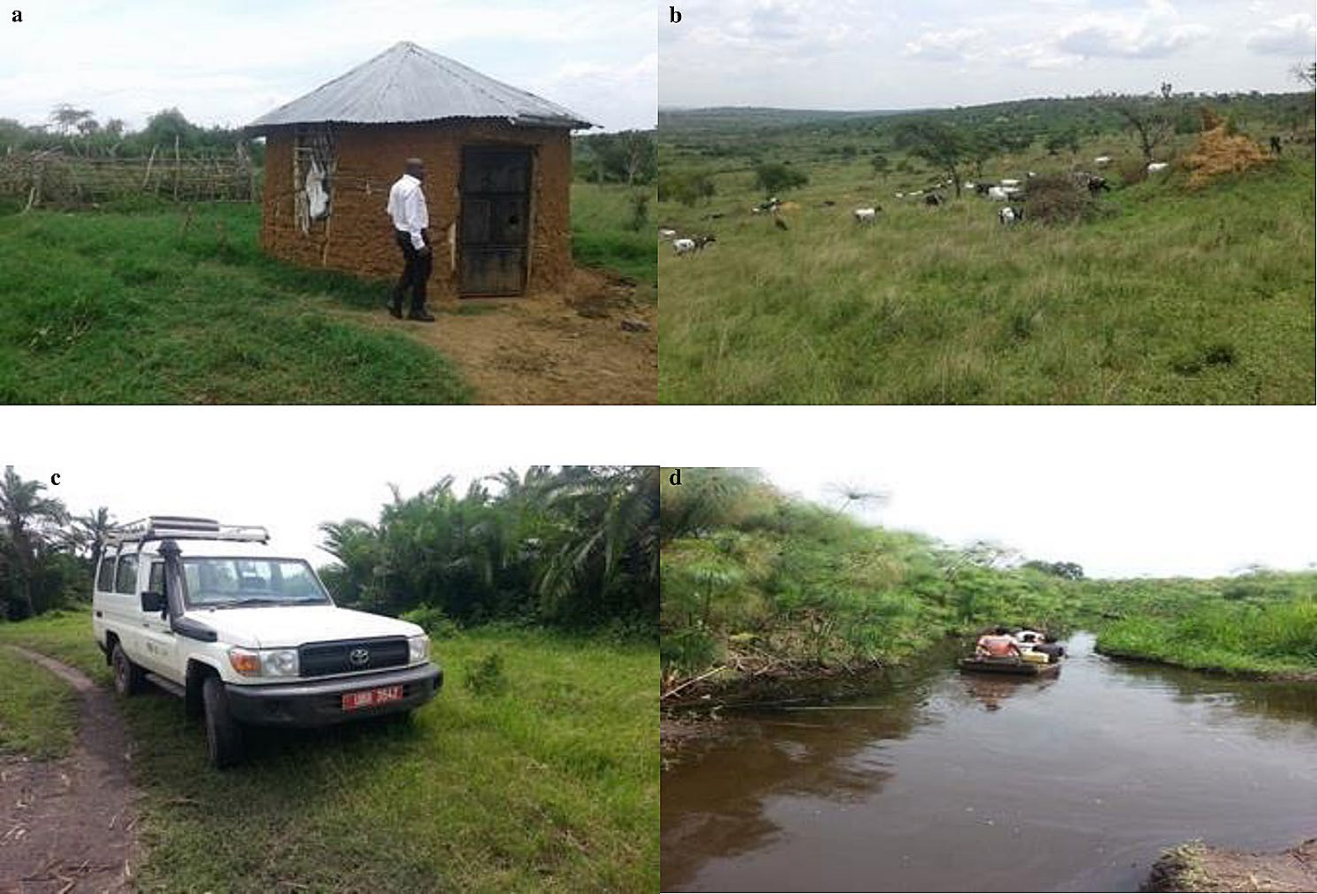
Pictures showing the remote settings from where cases were recruited. Upper left (**a**) – cattle kraal in the background close to residential house; upper right (**b**) - farm close to where one of the cases was recruited; lower left (**c**) – foot path to patient’s home that ended on a river in the lower right (**d**) beyond which villages are connected by canoe as the mean of transport

## Discussion

In this study, we investigated the mortality and morbidity among RVF confirmed cases reported to the UVRI and NRRT. Slightly more than half (53%) of all the confirmed acute cases between November 2017 and March 2020 died, a stark contrast to the absence of mortality among cases diagnosed in the country in 2016 and the 1960s. This mortality is higher than that reported by Nanyingi and colleagues [4], and more than two-fold higher than estimated in a recent meta-analysis [2]. Similarly, the proportion of RVF patients that required hospitalisation, the basis on which we defined severe disease [11] was 70%, out of whom 64% succumbed to the illness. The mortality was higher in district (80%) and regional referral hospitals (69%) compared to private hospitals (33%) and health care centre level III (50%). This severity of disease should concern us all as it is atypical of most RVF outbreaks. A review by Baba et al. in 2015 reported an increasing severity of RVF disease in endemic areas [14].

Patients die due to a delay in seeking health care, slow referral mechanisms, and initiating hospital based supportive care [15]. As illustrated in Fig. 6a - d, patients lived in remote locations some inaccessible by road during the rainy seasons when RVF outbreaks occur. In this study there was a delay of 7 days between onset of symptoms and laboratory confirmation of RVF despite a laboratory turn-around time of 24 hours. Such delays would have been avoided if rapid point-of-care diagnostics were readily available [16]. Nearly all (96%) hospitalised patients presented with pyrexia and haemorrhage at admission, out of whom 63% died. Haemorrhagic manifestations accounted for nearly 50% of death among RVF patients in some parts of Saudi Arabia [17], and dominated in Sudan [18] and Madagascar [19] outbreaks.

In this study, all patients who were above 44 years died. These findings contrast with those reported in Kenya where death occurred in patients much younger (21–30-year) and possibly related to greater exposure through male mandated pastoral duties with the animals [20]. The relationship between advanced age and mortality has been well characterised with viral infections and thought to be related to reduced adaptive immune responses as a result of natural age related involution of the thymus [21] as well as other comorbidities that affect the integrity of the immune system. In other RVF studies, mortality was associated with hospitalisation [20], HIV positive status [22], and RVF viral loads greater than 2.1 × 10^4^ viral particles/ml [23] during the first week since onset of symptoms. The high mortality in males mirrors the dominancy of the same gender in domestic animal care duties, trade, and veterinary service delivery. In this outbreak over 90% of RVF cases were males and the only sex enrolled in the follow-up study. Other potentially risky occupations included herding, farming, assisting animal birth, touching sick and dead animals, slaughtering animals, nursing febrile patients, and death of family members from febrile illness. Most survivors self-reported consumption of milk, meat, and ghee, although two patients independently reported consumption of wild game (squirrel) and an animal fetus delivered by caesarean section. We did not ascertain the RVF infection status for the fetus, cow or other consumed products through the laboratory. Another study undertaken in animals from RVF outbreak areas will provide insight on the possible correlation between human and animal cases [8]. Fetuses potentially have high viral loads and patients who acquire infections as a result of assisting animal births and disposal of fetuses are likely to present with severe disease [24].

In Uganda, cases of RVF in the 1960s were mild and none required hospital admission [6]. The cases were detected in a village whose inhabitants did not keep livestock and near the location of the UVRI in Entebbe, then Mpigi district. This area is geographically distant from the current RVF outbreak locations. It is possible that earlier RVF virus studies with laboratory animals in the 1940s could have posed a risk to the surrounding communities through mosquito transmission, as there is evidence of accidental infections among laboratory workers at the time [25]. There was no reported human disease for nearly 50 years till 2016 when three cases occurred in the southwestern district of Kabale [26]. These presented features of severe disease such as hepatitis, haemorrhage, and hospitalisation but there was no death. Between November 2017 and March 2020, over 50% of the cases died, 70% hospitalised, and presented with hepatic, renal, visual, haemorrhage, the severe forms of disease. It is possible that this increase in cases and disease severity is apparent due to increased awareness, surveillance and detection, and access to health care. Conversely, the increase in cases could be related to changes in climatic conditions such as heavy rains and flooding with subsequently increased breeding of different mosquito vectors, and/or from increased human activity that link the sylvatic and domestic RVF transmission cycles such as grazing cattle in gazetted wild game reserves. The communities of three districts from which confirmed cases were identified share an unfenced border with the Lake Mburo game park. In addition, the high human death from RVF could be related to unknown underlying co-morbidities in patients, and/or depict a trend of possibly worsening disease presentation over the years. Some phylogenetic studies have shown evolutionary changes in the RVF genome that mirror the virulence and severity of disease in humans [14]. Using next generation sequencing, two viral lineages: the Kenya 2 and the K/E lineages caused this outbreak [8]. The S-segment sequences for the K/E lineage had homology with the Beijing strain isolated in a patient from Angola in 2016. The Beijing-Angola patient presented with a life-threatening RVFV mediated cytokine release syndrome and the virus had reassortment of the L and M segments from Lineage E and the S segment from lineage A [27]. Lineage A viruses were associated with over 200,000 human cases and 598 deaths in Egypt in 1977–1979 [4, 28], and are known to be highly pathogenic and fatal in Wistar-furth rats, thus are a major virulence factor [28]. The Kenya-2 lineage contained sequences similar to viruses that caused outbreaks in Kenya 2006/2007 [8]. Although nucleotide sequences belonging to this lineage are highly conserved with minimal sequence variation in the order of 0.69%, 0.7%, and 0.86% for L, M, and S segments respectively, they are known to have undergone distinct RNA segment mutations that resulted to between 0.15 and 2.2% amino acid substitutions in the nucleoprotein, NSm, NSs, Gn, Gc, and RNA polymerase proteins [29]. The highest rate and uniqueness of mutations occurred in the Gn protein which is responsible for cell attachment, fusion and entry, and is the main target for neutralising antibodies. These substitutions, though rare could be responsible for the viral virulence of RVF as occurrence of minor changes in amino acid sequences of other arboviruses such as West Nile virus [30] and Chikungunya [31] have resulted in viruses with increased virulence and transmission effectiveness respectively. The occurrence of amino acid alterations in the viral strains within this outbreak require further evaluation. This could aid our understanding of the increased frequency and severity of disease as well as designing appropriate interventions. One major challenge that might be encountered is the occurrence of outbreaks big enough and persisting for quite a long time to conduct effectiveness studies. Our epidemiological cluster tree showed cases sparsely distributed over wide geographical areas with many villages or districts having a single case. This is not typical of case distribution during outbreaks and may be due to reporting bias but also, majority of RVF cases present with subclinical disease. Therefore epidemiological, and interventional studies may need to adapt to designs centred around confirmed cases.

Gastro-intestinal (GI) manifestations were the most common presentation among survivors. The dominancy of GI syndrome presentation was reported in one part of Saudi Arabia [32] and is characterised as a mild form of disease [11]. We observed a persistence of symptoms such as fever or feverishness, headache, muscle pain, joint pains and GI symptoms over 2–4 months and we believe this is unlikely to be a continuation of the acute illness. RVF symptoms lasting 4 to 120 days have also been reported in Tanzania [22]. This phenomenon has been observed in relation with other viral infections including severe acute respiratory syndrome corona virus 2 (SARS-CoV-2) [33, 34] and described as post viral exhaustion syndrome (PVES) [35], a form of chronic fatigue syndrome (CFS) or myalgic encephalomyelitis (ME) [36]. The occurrence of this syndrome has not been previously described in relation with RVF. Studies of CFS/ME following viral infections have provided some compelling pathophysiological insight, that the cytokine storm [34, 37] and mitochondrial/oxidative stress [38] during acute infection mediate the persistence of symptoms. The high cytokine milieu causes mitochondrial damage, an organelle responsible for 90–95% of body energy production [38]. The defects in cellular energy production manifest in patients as fatigue, cognitive impairment, myalgia, feverishness, or other systemic manifestations, against a backdrop of normal full blood count (FBC).

Acute RVF causes a pancytopenia characterised by leukopenia, anaemia, thrombocytopenia, and neutropenia in humans [17, 26, 32]. Long-term haematological follow-up studies have not been conducted among survivors. We followed up survivors to investigate whether haematological parameters were deranged in the long-term by the acute infection. Our results show that all FBC parameters recovered to normal levels by three months and were maintained during the one-year follow-up. The absence of a long-term bone marrow suppression is reassuring for survivors and in vaccine development. Other viral infections are known to impair the reconstitution of long-term hematopoietic stem cells (LT-HSC) [39]. The bone marrow niche hosts long lived plasma cells which persistently produce high affinity antibodies following infection or vaccination [40].

Elevation of liver enzymes such as AST, ALT, and ALP is a characteristic feature of RVF in humans [17, 32]. The liver is the main target organ for RVF, and causes varying degrees of hepatic necrosis. Hepatic regeneration starts within 5 days post infection in mice [41], however, long-term liver recovery has not been reported in humans. In our study, liver enzymes returned to normal within three months and were maintained throughout follow up. This study used cross-sectional, retrospective and prospective approaches to data collection. Medical history obtained months after the acute illness is prone to recall bias. The longitudinal follow-up of survivors has provided insight into possibilities of RVF associated CFS/ME and assurance that patients regain normal FBC and liver enzyme levels in the long-term. We had limited clinical and laboratory data during the acute phase of the illness as hospital records were not accessed, and symptoms of PVES/CFS/ME were not systematically solicited thus our inference on PVES is suspected or incidental. The lack of negative controls or infection status of the potential sources of risk limits our inferences on RVF risk factors as these could as well be normal levels of exposure to these disease determinants in the general population. Finally, the study had a small sample size and most likely captured patients with severe disease that presented to the health care system.

In conclusions, RVF causes high mortality, acute and long-lasting morbidity in patients that present to the health care system with the disease. Patients report late for care when the role of antivirals to maintain life would be limited, therefore, point-of-care diagnostics, serum markers of prognosis and potential biologics should be identified to improve patient care. One-health combined interventions should be developed to improve environmental and livestock surveillance, prevent infections and promptly detect outbreaks. These and more measures would be composite in curbing the sparingly known disability adjusted life years (DALYs) associated with RVF.

### Electronic supplementary material

Below is the link to the electronic supplementary material.

Supplementary Material 1. Additional file 1. DOC (Microsoft Word). Standard case definition for reporting suspected Rift Valley fever cases from the health facility to the district health office in Uganda

## Data Availability

The data that support the findings of this study are not openly available due to reasons of sensitivity and are available from the corresponding author upon reasonable request. Data are located in controlled access data storage at the MRC/UVRI and LSHTM Uganda Research Unit.

## Abbreviations

ALP: Alkaline phosphatase
ALT: Alanine aminotransferase
AST: Aspartate aminotransferase
CFS: Chronic fatigue syndrome
D: District
DALYs: Disability Adjusted Life Years
ELISA: Enzyme Linked Immunosorbent Assay
FBC: Full blood count
Gc: glycoprotein ectodomain encoded by carboxy-terminal sequences
GI: Gastrointestinal
Gn: glycoprotein ectodomain encoded by amino-terminal sequences
IgG: Immunoglobulin G
IgM: Immunoglobulin M
LFTs: Liver function tests
LSHTM: London School of Hygiene and Tropical Medicine
LT-HSC: Long-term hematopoietic stem cells
ME: Myalgic encephalomyelitis
MeSH: Medical subject heading
NRRT: National Rapid Response Teams
NSm: Non-structural protein medium
NSs: Non-structural protein small
P: Parish
PBMCs: Peripheral blood mononuclear cells
PCR: Polymerase chain reaction
PVES: Post viral exhaustion syndrome
RNA: Ribonucleic acid
RRH: Regional referral hospital
RVF: Rift Valley fever virus
RVFV: Rift Valley fever virus
S: subcounty
SARS-CoV-2: Severe acute respiratory syndrome corona virus 2
UK: United Kingdom
UNCST: Uganda National Council for Science and Technology
UVRI: Uganda Virus Research Institute
UVRI REC: Uganda Virus Research Institute Research and Ethics committee
V: Village
VHFs: Viral haemorrhagic fevers
