# Supplementary material for: Severe morbidity and hospital-based mortality from Rift Valley fever disease between November 2017 and March 2020 among humans in Uganda
Source: Virol J. 2024 May 3;21:104. doi: 10.1186/s12985-024-02377-z (PMC11069174; doi:10.1186/s12985-024-02377-z)
Supplement: Supplementary file 1 — Supplementary Material 1. Additional file 1. DOC (Microsoft Word). Standard case definition for reporting suspected Rift Valley fever cases from the health facility to the district health office in Uganda [file 12985_2024_2377_MOESM1_ESM.docx]

**Additional file 1:** Standard case definition for reporting suspected Rift Valley fever cases from the health facility to the district health office in Uganda ([1](#_ENREF_1)).

|  |
| --- |
| **Suspected case**  **Early disease**   - Acute febrile illness (axillary temperature >37.5 ºC or oral temperature of >38.0ºC) of more than 48 hours duration that does not respond to antibiotic or antimalarial therapy, and is associated with: - Direct contact with sick or dead animal or its products **AND / OR**: - Recent travel (during last week) to, or living in an area where, after heavy rains, livestock die or abort, and where RVF virus activity is suspected/confirmed **AND / OR:** - Abrupt onset of any 1 or more of the following: exhaustion, backache, muscle pains, headache (often severe), discomfort when exposed to light, and nausea/vomiting **AND / OR**: - Nausea/vomiting, diarrhoea, OR abdominal pain with 1 or more of the following:   - Severe pallor (or Hb < 8 gm/dL)   - Low platelets (thrombocytopenia) as evidence by presence of small skin and mucous membrane haemorrhages (petechiae) (or platelet count < 100x109 / dL)   - Evidence of kidney failure (oedema, reduced urine output) (or creatinine > 150 mol/L) **AND / OR**:   - Evidence of bleeding into skin, bleeding from puncture wounds, from mucous membranes or nose, from gastrointestinal tract and unnatural bleeding from vagina **AND / OR:**   - Clinical jaundice (3-fold increase above normal of transaminases)   **Late stages of diseases or complications (2-3 weeks after onset)**   - Patients who have experienced, in the preceding month a flu-like illness, with clinical criteria, who additionally develop the following: - CNS manifestations which resemble meningo-encephalitis **AND/OR** - Unexplained visual loss **OR** - Unexplained death following sudden onset of acute flu-like illness with haemorrhage, meningo-encephalitis, or visual loss during the preceding month.   **Confirmed case**  Any patient who, after clinical screening, is positive for anti-RVF IgM ELISA antibodies (typically appear from fourth to sixth day after onset of symptoms) or tests positive on Reverse Transcriptase Polymerase Chain Reaction (RT-PCR). |

Reference

1. MOH U. Case definitions and epidemic thresholds for integrated disease surveillance and response (IDSR): A working guide for health workers. URL <https://www.afro.who.int/sites/default/files/2021-09/2_Uganda%203rd%20IDSR%20Tech%20Guideline_PrintVersion_10Sep2021.pdf>. Accessed 09 January 2023.
